# Welding of Advanced Aluminum–Lithium Alloys: Weldability, Processing Technologies, and Grain Structure Control

**DOI:** 10.3390/ma19040738

**Published:** 2026-02-14

**Authors:** Qi Li, Qiman Wang, Yangyang Xu, Peng Sun, Kefan Wang, Xin Tong, Guohua Wu, Liang Zhang, Yong Xu, Wenjiang Ding

**Affiliations:** 1 National Engineering Research Center of Light Alloy Net Forming and State Key Laboratory of Metal Matrix Composites, School of Materials Science and Engineering, Shanghai Jiao Tong University, Shanghai 200240, Chinaliangzhang08@sjtu.edu.cn (L.Z.); wjding@sjtu.edu.cn (W.D.); 2 Special Welding Center of Technology Innovation, Shanghai Aerospace Equipment Manufacturer Co., Ltd., Shanghai 200245, China13917011056@163.com (Y.X.)

**Keywords:** welding of Al-Li alloys, grain structure, equiaxed grain zone (EQZ), columnar grain, weldability

## Abstract

Aluminum–lithium (Al-Li) alloys are extensively employed in aerospace and space structures because of their low density, high specific stiffness, and excellent fatigue resistance. However, welding of these alloys remains challenging, since the joints typically exhibit unique microstructural features, including equiaxed grain zones (EQZ) along the fusion boundary and coarse columnar grains in the fusion zone, which degrade mechanical performance and increase susceptibility to cracking. This review provides an overview of the generational evolution of Al-Li alloys and their associated weldability, highlights the advantages and limitations of major welding processes, such as laser, arc, and hybrid techniques, and systematically examines the formation mechanisms of EQZ, columnar grains, and equiaxed grain bands. Various strategies for microstructural control are compared, including filler design, pulsed current, and external-field-assisted welding. Special attention is given to grain refinement achieved through heterogeneous nucleation, dendrite fragmentation, and columnar-to-equiaxed transition. Finally, prospects for advanced microstructural control strategies are discussed, with the goal of achieving high-quality welds for next-generation lightweight structural applications.

## 1. Introduction

Aluminum–lithium (Al-Li) alloys are a class of lightweight, high-performance materials produced by adding Li to conventional aluminum alloys [1,2,3,4,5,6]. Each 1 wt.% addition of Li has been reported to reduce the alloy density by approximately 3% while increasing the elastic modulus by about 6% [7,8]. In the aerospace sector, Al-Li alloys are widely employed in critical components subjected to cyclic loading, such as fuselage skins and wings, owing to their excellent fatigue resistance and high damage tolerance, which together enhance flight safety [9,10,11]. In the space sector, Al-Li alloys are also used in the fabrication of fuel tanks and other structural parts [12,13]. Compared with conventional Al-Cu alloy tanks, Al-Li fuel tanks are lighter and more resistant to deformation, achieving a weight reduction of about 15%, thereby lowering launch costs and improving payload capacity [14]. With the progressive engineering application of Al-Li alloys, extensive attention has been directed toward their forming technologies, including additive manufacturing [15] and welding [16].

**Figure 1:**
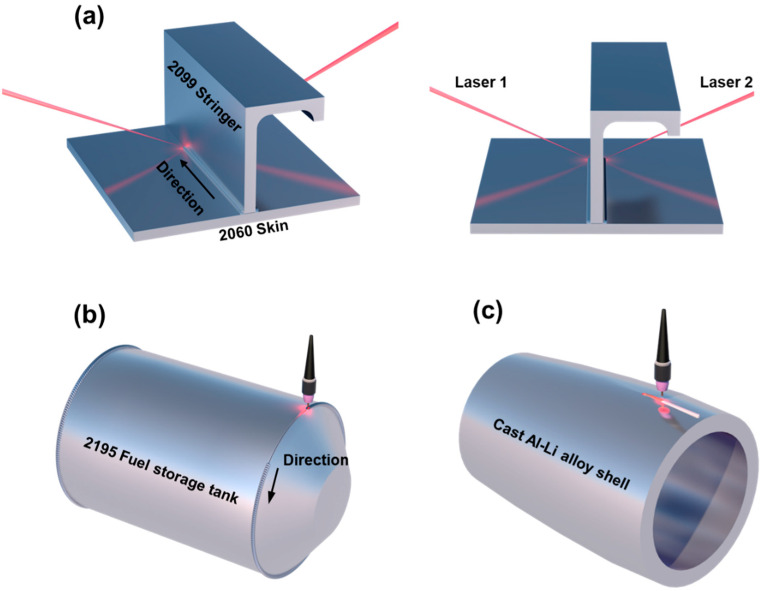
Applications of welding in Al-Li alloys: (**a**) fabrication of skin-stringer T-joints in aircraft using dual laser-beam synchronous welding; (**b**) joining of the tank dome and shell in fuel storage tanks by TIG welding; (**c**) repair welding of Al-Li alloy castings by TIG welding.

Welding is a key technology in the fabrication of Al-Li alloy components, and most currently applied Al-Li structures inevitably rely on it [17,18,19,20]. In the manufacturing of aircraft skin-stringer structures, Airbus developed a dual laser beam welding (DLBW) process to replace traditional riveting, enabling the transition from assembled structures to integral structures [21,22], as illustrated in Figure 1a. This DLBW technique has been shown to reduce structural weight by 5–10% and manufacturing cost by about 15% without compromising strength or fatigue life [23,24]. Tungsten Inert Gas (TIG) welding has the longest history and the highest level of maturity in the fabrication of aerospace propellant tanks, where it is commonly used to join the tank dome and the shell, as shown in Figure 1b [25,26]. More recently, TIG welding has also been reported for repairing casting defects in large Al-Li alloy castings with high Li contents, as illustrated in Figure 1c [27].

The most distinctive feature of welded joints in Al-Li alloys, compared with other metallic welds, is their unique grain structures. In the partially melted zone (PMZ), extremely fine equiaxed grains with diameters of approximately 5 μm are distributed along the fusion boundary, forming a fine-grain band with a width of several tens of micrometers [28,29,30,31,32]. However, these fine grains do not contribute positively to the tensile performance of the joint; instead, they constitute the weakest region of the weld, where microcracks are frequently observed [33,34]. In the fusion zone (FZ), coarse columnar grains with relatively large sizes and high-volume fractions are commonly found [35,36,37]. In welding processes characterized by steep thermal gradients, such as laser welding, the FZ is almost entirely composed of columnar grains several hundred micrometers in size [36,38]. These coarse columnar grains not only cause anisotropic mechanical properties but also create narrow interdendritic liquid channels that markedly increase solidification cracking susceptibility, a critical concern for Al-Li alloys, which already possess poor weldability [38,39,40]. Previous studies have frequently reported the occurrence of fine-grain bands in the welds of Al-Li alloys, while others have emphasized the detrimental role of coarse columnar grains in the FZ. However, the findings remain fragmented and sometimes contradictory, reflecting the complexity of grain structural evolution under different welding conditions. Therefore, despite considerable progress in understanding the weldability and microstructural evolution of Al-Li alloys, persistent challenges such as EQZ formation, coarse columnar grains, and defect susceptibility remain unresolved.

In this review, the weldability, welding processes, and grain structure characteristics and control of Al-Li alloy joints are comprehensively summarized. While previous reviews have provided valuable insights into the weldability of Al-Li alloys, continued alloy development and advances in welding technologies call for an updated overview that reflects recent progress in this field. In particular, this work highlights the evolution of grain structures and microstructural control strategies, with renewed attention not only to the EQZ near the fusion line but also to the columnar grain zone (CGZ) at the weld center, which has received limited discussion in earlier reviews. By integrating both aspects, this review provides a more complete understanding of the fusion zone microstructure, offering guidance for the reliable application of Al-Li alloys in next-generation aerospace structures.

## 2. Generational Evolution and Weldability of Al-Li Alloys

Al-Li alloys have undergone three stages of development, commonly referred to as first-, second-, and third-generation systems [41,42,43]. Each generation possesses distinct alloy chemistries and welding characteristics that define their applicability in aerospace structures. The following section provides a comparative overview of their compositions, weldability, and representative applications.

The first-generation Al-Li alloys belong to the Al-Mg-Li system, with alloy 1420 being the most representative. This alloy was employed in a modified MiG-29M fuselage, where welded joints replaced riveted structures and achieved a weight reduction of approximately 10% [44]. The fuel tanks of the MiG-29 aircraft were also fabricated by welding 1420 alloy and remain in service today [44]. In terms of composition, these alloys contain relatively high amounts of Li and Mg together with minor additions of Sc and Zr. The presence of Mg enhances resistance to solidification cracking by reducing grain boundary segregation and promoting grain coalescence during solidification [45]. However, the high Li content markedly increases porosity sensitivity. For instance, Orishich et al. [46] reported that laser-welded 1420 joints exhibited extensive porosity, whereas Wang et al. [47,48] demonstrated that removing surface oxides and hydroxides could effectively mitigate this defect. Based on this alloy system, the developed Al-Mg-Li-Ag-Sc-Zr composition has been demonstrated to be suitable for laser powder bed fusion additive manufacturing [49]. The primary Al_3_(Sc,Zr) phases in the microstructure effectively refine grains and work in synergy with submicron T-Mg_32_(Al,Ag)_49_ and nanoscale S_1_-Al_2_MgLi phases to strengthen the alloy. This results in a tensile strength exceeding 400 MPa while maintaining an elongation of 12.5%, demonstrating excellent adaptability to the additive manufacturing process.

To address these shortcomings, second-generation Al-Li alloys were developed based on the Al-Cu-Li system. They display higher strength, improved fatigue performance, and superior fracture toughness while maintaining low density [50,51,52,53]. Their Cu content typically ranges from 1 to 2 wt.%, which corresponds to the composition range most prone to solidification cracking in Al-Cu alloys [54], making alloys such as 2090, 1430, and 1440 highly susceptible. In addition, their Li content above 2 wt.% results in severe hydrogen porosity, as observed in alloy 8090 with 2.5 wt.% Li [55,56,57,58]. Consequently, second-generation alloys generally exhibit poor weldability. To improve this, alloy 1460 was developed with approximately 3 wt.% Cu, which increases the amount of eutectic liquid during solidification and compensates for shrinkage cavities [59], thereby enhancing resistance to solidification cracking [60,61]. Moreover, trace Sc additions refine α-Al grains, suppress columnar grain growth under steep thermal gradients, and further improve weldability [62].

Building on the limitations of the second generation, which included poor weldability, anisotropy, and insufficient thermal stability, a series of third-generation Al-Li alloys was introduced. Their higher Cu/Li ratio markedly improves weldability, while trace additions of Ag and Mg promote the precipitation of strengthening phases, thereby enhancing both strength and toughness [5,63]. Reynolds and Martin Marietta jointly developed the Weldalite™ 049 series (Martin Marietta Laboratories, Baltimore County, MD, USA), which achieved a tensile strength exceeding 690 MPa and an elongation of 5.3% [64]. This series includes AA2094, AA2095, 2096, 2195, and 2197, among which alloy 2195 was successfully employed in space shuttle propellant tanks. The replacement of alloy 2219 with 2195 resulted in a 30% increase in strength and a 3.4-ton reduction in weight, thereby increasing payload capacity [65,66]. Based on Weldalite™ 049, the addition of 0.5 wt.% Zn produced Weldalite™ 210 (Reynolds Metals Company, Richmond, VA, USA), in which Zn refined T1, θ′, and δ′ precipitates, further increasing the yield strength (731 MPa) and tensile strength (758 MPa), although at the cost of reduced ductility (2.8%) [66]. In parallel, Constellium (Chicago, IL, USA) and Alcan (Montreal, QC, Canada) developed the Airware™ series (AA2196 and AA2198), which have been applied in A380E and A350C aircraft structures [67]. A summary of the composition, weldability, microstructure, and defects in Al-Li alloy welds across various developmental stages is presented in Table 1.

## 3. Welding Processes for Al-Li Alloys

Welding processes are critical to the fabrication and repair of Al-Li alloys, as they directly influence weld morphology, grain structure, and defect formation. Among these processes, laser welding and arc welding are the most widely applied and extensively investigated methods. Laser welding provides high precision and productivity but is highly susceptible to porosity and cracking due to steep thermal gradients. In contrast, arc welding techniques such as TIG welding and variable polarity plasma arc welding (VPPA) produce stable joints and enable effective oxide removal, although they are generally less productive. A comparative assessment of these processes is therefore essential for optimizing the weldability of Al-Li alloys in aerospace applications.

### 3.1. Laser Welding

**Figure 2:**
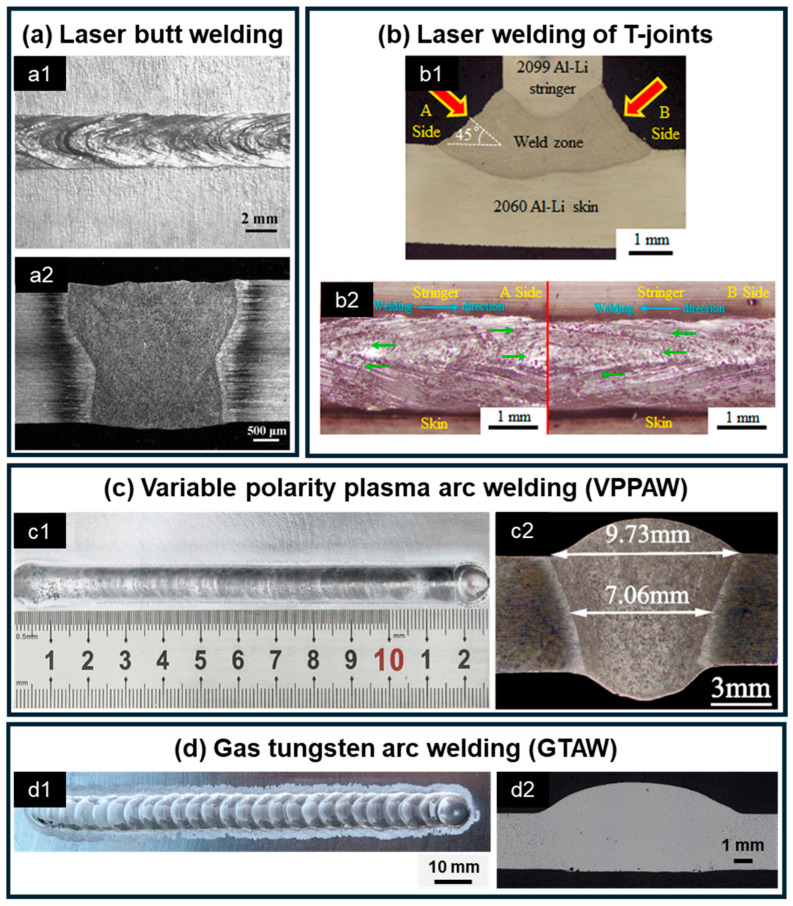
Weld formation of Al-Li alloys using different fusion welding methods: (**a**) laser butt welding: (**a1**) surface morphology and (**a2**) weld cross-section [69]; (**b**) laser welding of T-joints: (**b1**) surface morphology and (**b2**) weld cross-section [70]; (**c**) variable polarity plasma arc welding (VPPAW): (**c1**) surface morphology and (**c2**) weld cross-section [71]; (**d**) tungsten inert gas (TIG) welding: (**d1**) surface morphology and (**d2**) weld cross-section [27].

Laser welding is a highly efficient and precise joining technique that employs a high-energy-density laser beam as the heat source. Its principal advantages include a narrow heat-affected zone, high welding precision, and the ability to achieve full penetration in thick plates. Consequently, this method is widely used for welding Al-Li alloy components that require high productivity and automation, including butt welding of thick tank lids and shells as well as dual-beam laser welding of aircraft skin-stringer structures. In terms of weld morphology, as shown in Figure 2a, the surface of Al-Li alloy laser-welded butt joints is characterized by a narrow fusion width and large penetration depth. According to Liu et al. [68], this cross-sectional profile results from the combined effects of metal vapor recoil pressure and the Marangoni effect, which drive molten metal on both the upper and lower surfaces of the weld pool outward, while convection in the central waist region is weakest, thereby producing the characteristic “wide-wide-narrow” shape. The characteristic narrow heat-affected zone (HAZ) produced by this high-energy-density process typically measures approximately 0.5 mm in Al-Li alloys [36,68]. However, this region experiences grain coarsening and precipitate coarsening/dissolution due to the thermal cycle, leading to noticeable softening. Microhardness measurements reveal that the HAZ exhibits values 20–50 HV lower than the wrought base metal (BM) but remains 40–80 HV higher than the as-cast FZ.

**Figure 3:**
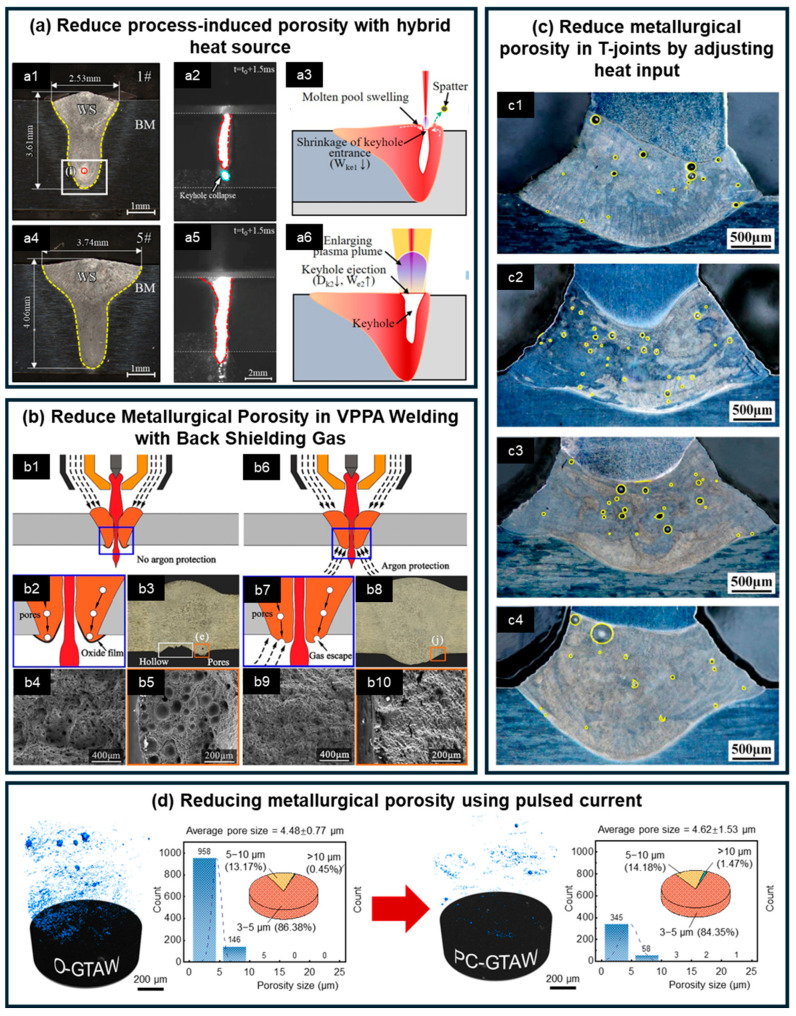
Welding Porosity Characteristics and Solutions of Al-Li Alloys under Different Welding Methods [27,72,73,75]. (**a**) Porosity formation in laser welding and the role of hybrid heat sources: (**a1**) and (**a4**) show weld cross-sections; (**a2**) and (**a5**) present keyhole morphologies; (**a3**) and (**a6**) illustrate the keyhole interaction mechanisms under conventional and hybrid heat sources, respectively. (**b**) Porosity formation in VPPA welding and the effect of back shielding gas: (**b1**–**b5**) depict the porosity formation mechanism and corresponding fracture morphologies without back shielding gas; (**b6**–**b10**) illustrate the mechanism by which back shielding gas promotes bubble escape and the associated fracture morphologies. (**c**) Porosity in laser welding of T-joints and the influence of heat input, with (**c1**–**c4**) corresponding to progressively increasing heat input. (**d**) Effect of pulsed current on porosity formation.

**Figure 4:**
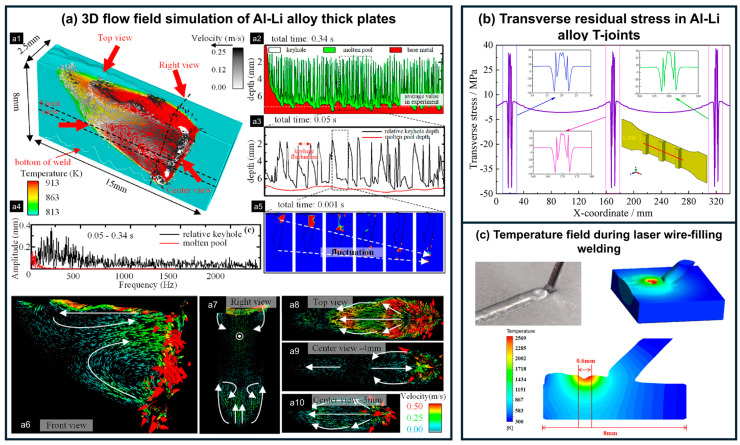
Numerical simulations of Al-Li alloy laser welding: (**a**) 3D flow field of the molten pool in thick plates [77], comprising (**a1**) a schematic of the 3D flow field, (**a2**–**a5**) oscillation depth, frequency and characteristics of the molten pool, and (**a6**–**a10**) flow field distributions from different viewing angles; (**b**) residual stress in T-joints [78], and (**c**) temperature field distribution during wire-filling welding [79].

Laser welding of Al-Li alloys is prone to serious defects. For T-joints (Figure 3c), the surface morphology resembles that of butt joints; however, the molten zone is generated by the interaction of two overlapping pools created by dual laser beams. This interaction produces more complex flow behavior in the molten pool and a higher sensitivity to porosity. In addition, structural constraints hinder bubble flotation due to the presence of stringers, resulting in an increased number of pores, as reported by Enz et al. [67]. Chen et al. [72] investigated porosity formation in dual-beam laser-welded 2060/2099 Al-Li T-joints and showed that the joint configuration impedes bubble escape, while the evaporation of low-melting-point elements such as Mg and Zn further aggravates metallurgical porosity (Figure 3c). Moreover, the presence of Li decreases the surface tension of the melt, which reduces keyhole stability and in severe cases can trigger keyhole-induced porosity. To mitigate this problem, Zhao et al. [73] proposed a fiber-diode laser coaxial hybrid heat source, which significantly improved molten pool flow and keyhole stability, thereby reducing the occurrence of porosity and collapse defects. As shown in Figure 3a, Zhao et al. [73] demonstrated that this hybrid source widened the keyhole opening, prevented its closure by molten metal backflow, and thus avoided keyhole-induced porosity. Furthermore, the steep temperature gradients inherent to laser welding result in high strain rates during solidification, which, together with the aforementioned material factors, make cracking a critical concern in Al-Li alloys. Regarding mitigation strategies, as illustrated in Figure 4a, Enz et al. [74] evaluated the effects of preheating, preloading, laser spot diameter, process parameters, and shielding gas on solidification cracking in AA2198 Al-Li alloy. Their results indicated that all of these measures reduced crack susceptibility. Among them, the most effective approach was the use of low laser power and low welding speed, which produced a smaller molten pool, decreased the temperature gradient, and significantly reduced solidification shrinkage stresses in the weld. The high susceptibility of Al-Li alloys to porosity and cracking has also been documented in additive manufacturing processes [75,76]. Xu et al. [75] demonstrated that during selective laser remelting of Al-Cu-Li alloys, increasing the heat input from 48.61 J/mm^3^ to 100 J/mm^3^ reduced molten pool tension and enhanced its fluidity. This improvement promoted sufficient liquid phase diffusion at elevated temperatures, effectively minimizing defects in the AM process and increasing the deposition layer density from approximately 94.1% to 97.8%. In another study, Xue et al. [76] produced AA2196 alloy via wire-arc additive manufacturing and observed that T6 heat treatment coarsened large pores in the deposited layer (reaching up to 107 μm). However, by applying 42% hot deformation, the porosity was significantly reduced from 1% in the as-deposited state to 0.01%.

To achieve a deeper understanding of the characteristics of laser welding in Al-Li alloys, extensive numerical simulations have been performed. One of the key roles of numerical simulation is to provide an intuitive visualization of phenomena that are difficult to observe experimentally. In the field of Al-Li alloy welding, computational fluid dynamics is commonly employed to simulate weld pool flow behavior, where detailed information on molten metal convection and temperature distribution can be obtained-data that are challenging to capture experimentally. The grain structures in Al-Li alloy welds are highly complex; the phase-field method enables the reproduction of grain nucleation and growth during solidification and can further predict post-weld grain morphology and structure based on solute distribution and nucleation rate, thereby guiding the design of filler compositions and welding parameters. The finite element method provides reliable predictions of stress and deformation in welded structures. For the T-joint configurations commonly used in Al-Li alloys, this approach effectively predicts cold cracking and structural distortion, ensuring post-weld dimensional accuracy. Table 2 summarizes and compares the numerical simulation methods commonly used for Al-Li alloy welding, outlining their purposes and major research findings.

As shown in Figure 4a, Han et al. [36,77,80] simulated molten pool behavior during deep-penetration welding of thick plates and revealed that significant velocity differences existed across different regions of the pool, which strongly influenced the grain morphology of laser-welded Al-Li joints. Guo et al. [78] simulated the transverse residual stress in T-joints after laser welding (Figure 4b), while Liu et al. [79] investigated the thermophysical field during laser wire-filling welding (Figure 4c). These simulation studies provide valuable theoretical insights into the welding mechanisms of Al-Li alloys and offer important guidance for their engineering applications.

### 3.2. Arc Welding

Arc welding of Al-Li alloys primarily involves Tungsten Inert Gas (TIG) welding and Variable Polarity Plasma Arc (VPPA) welding, both of which employ an arc as the heat source in combination with tungsten electrodes and inert gas shielding. TIG welding is among the most widely used arc welding techniques, characterized by process simplicity, high weld quality, and strong adaptability in repair applications, as it allows precise operation in narrow or confined spaces [81,82]. Recent studies have also demonstrated the effectiveness of TIG in repairing high-Li-content cast Al-Li alloys, further highlighting its versatility [27]. In contrast, VPPA utilizes a constricted plasma arc with variable polarity, which provides deeper penetration, greater arc stability, and more efficient oxide removal [71,83]. Owing to these advantages, VPPA has been successfully applied to the welding of large Al-Li alloy propellant tanks. Both TIG and VPPA also benefit from cathodic cleaning effects, which effectively remove oxide films containing Li and Al from the workpiece surface. The weld morphology of Al-Li alloys produced by VPPA and TIG welding is shown in Figure 2c,d, respectively. VPPA readily achieves full penetration in 6 mm-thick Al-Li plates, resulting in a smooth and flat weld surface. In comparison, the cross-section obtained by TIG welding is generally similar to that of VPPA, but the weld surface displays distinct textures, which mainly originate from the solidification of filler droplets during wire feeding. Unlike the highly concentrated heat source in laser welding, the broader arc interaction in TIG and VPPA welding results in a significantly wider HAZ, typically measuring approximately 1.5 mm [18,71]. When joining wrought BM, the microhardness of the HAZ is only slightly lower (by about 20 HV) or comparable to that of the BM. However, in the case of as-cast BM, the HAZ tends to develop liquation cracks due to the presence of eutectic phases. This leads to a more pronounced softening effect, with the HAZ exhibiting lower hardness than both the BM and the FZ, thereby becoming the weakest region of the entire joint. To mitigate this risk, strict control of the heat input (not exceeding 3.85 kJ/mm [27]) is essential when welding as-cast materials.

Compared with laser welding, arc welding of Al-Li alloys is less sensitive to process-induced porosity, with most pores being metallurgical in origin. Cui et al. [75] significantly reduced porosity by applying back shielding gas to the underside of welded plates. As illustrated in Figure 3, the back shielding gas prevents the formation of an oxide film at the weld root, thereby facilitating the escape of pores from the FZ into the molten pool. Li et al. [27] minimized porosity and microcracks in cast Al-Li alloys using a pulsed current technique. This effect was attributed to the periodic switching between high and low currents, which caused the molten pool to continuously expand and contract, maintaining a state of oscillation. The remelting provided bubbles with an additional opportunity to escape from the molten pool, while the oscillation further promoted bubble release [84]. Aside from welding defects, Li et al. [27] also investigated the distribution of solute Li in TIG welds under different parameters, specifically by increasing the welding current from 110 A to 150 A and the pulse frequency from 10 Hz to 1000 Hz. Their results indicated that the high welding current of 150 A led to the evaporation loss of Li. In contrast, the pulsed current process promoted a more uniform distribution of Li, effectively mitigating its intragranular segregation.

Table 3 summarizes and compares the primary welding processes for Al-Li alloys, including their joint efficiency, porosity control, advantages, and limitations. From a practical perspective, laser welding offers high production efficiency, excellent joint-fit precision, and a high degree of automation, making it suitable for joining thick Al-Li alloy plates and applications requiring tight dimensional control. However, due to the presence of keyhole-related defects, TIG and VPPA welding are preferred in situations demanding high defect tolerance. Their cathodic cleaning effect and relatively low heat input help suppress porosity and cracking, while lower equipment costs further enhance their economic feasibility. Although arc welding exhibits lower production efficiency, its superior operability allows the welding of curved or internal surfaces and complex structures, making it indispensable for repair and rework operations.

## 4. Microstructural Characteristics and Control Strategies in Al-Li Welds

**Figure 5:**
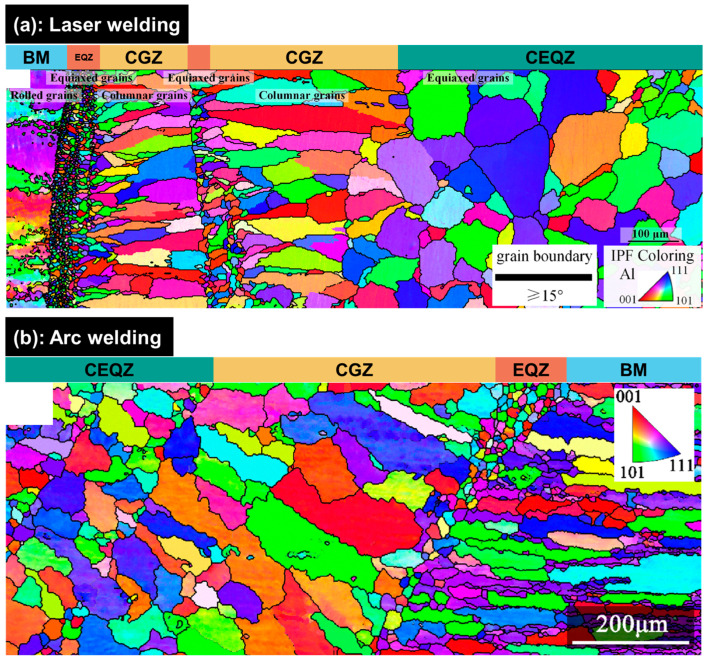
Typical grain structures of Al-Li alloy weld joints produced by different fusion welding methods [83,86] (CEQZ: central equiaxed grain region; EQZ: equiaxed grain zone; CGZ: columnar grain zone; BM: base metal): (**a**) laser welding; (**b**) arc welding.

Figure 5 presents the EBSD maps of Al-Li alloy weld joints produced by two mainstream fusion welding methods, illustrating the typical grain structure characteristics of these welds. The grain structure consists of the BM, the equiaxed grain zone (EQZ), the columnar grain zone (CGZ), and the central equiaxed grain zone. The following sections provide a detailed description of the EQZ and CGZ, the two microstructural features that most strongly influence the performance of Al-Li alloy welds, with particular attention to their characteristics, formation mechanisms, and possible control strategies.

### 4.1. Equiaxed Grain Zone (EQZ)

**Figure 6:**
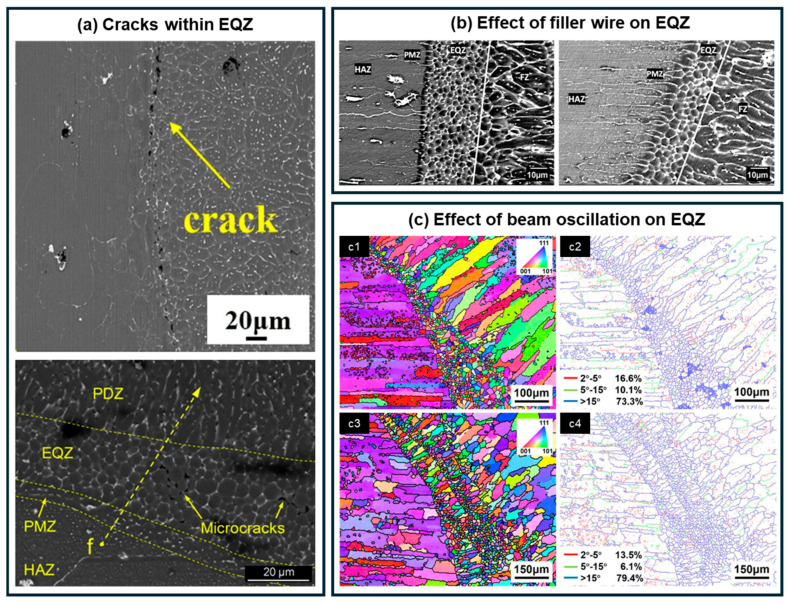
Hazards associated with the equiaxed grain zone (EQZ) in Al-Li alloy welds and corresponding mitigation strategies: (**a**) increased crack susceptibility of the EQZ [33,34]; (**b**) reduction in EQZ width through the application of filler wire [91]; (**c**) modification of EQZ characteristics by beam oscillation [92], with (**c1**,**c2**) showing the case without laser beam oscillation and (**c3**,**c4**) illustrating the condition under a ′8′-shaped laser beam oscillation.

The EQZ microstructure represents a distinctive feature of Al-Li alloys, distinguishing them from other metallic systems. This narrow band, composed of extremely fine grains (~5 μm), is typically located along the fusion line. Initially, it was suggested that the EQZ formed through recrystallization of the BM in its rolled condition under the thermal cycle imposed by welding. This hypothesis was supported by the equiaxed, non-dendritic grain morphology and the similarity in grain size between the EQZ and BM subjected to comparable thermal exposure [15]. However, this recrystallization-based explanation seems less persuasive, as comparable EQZ structures have rarely been reported in the welding of other rolled Al alloys. In contrast, Gutierrez et al. [87] provided compelling evidence that the EQZ originates from heterogeneous nucleation on particles such as Al_3_(Zr,Li) and Al_3_Zr present in the BM. They showed that alloys without Zr do not form an EQZ after welding, whereas alloys containing Zr do. Moreover, when comparing solution-treated BM (containing Al_3_Zr and Al_3_(Zr,Li) precipitates) with as-cast BM (without such precipitates), EQZ was observed only in the solution-treated material. These systematic results strongly validate the heterogeneous nucleation mechanism as the dominant explanation for EQZ formation. These high-melting-point particles remain undissolved near the fusion line due to the lower thermal exposure in that region, thereby promoting nucleation and the formation of the EQZ. In contrast, within the higher-temperature FZ, these particles dissolve, eliminating heterogeneous nucleation sites and resulting in the absence of EQZ. Lippold et al. [88] reported, during Varestraint testing of alloys 2094 and 2195, that solidification cracks propagated preferentially along the EQZ rather than along the weld centerline, a behavior that is atypical for most alloys. Similarly, tensile tests have frequently revealed crack propagation along this zone [33]. The susceptibility of the EQZ to cracking is likely attributable to insufficient strengthening phases caused by intergranular segregation [89], as well as an inherent propensity for solidification cracking (Figure 6a) [35].

Current research has focused on minimizing or eliminating the EQZ microstructure in Al-Li alloy welds. However, this goal remains challenging due to the unique characteristics of the EQZ, which forms in the partially melted zone adjacent to the fusion line. This region exhibits high melt viscosity and poor fluidity [90]. According to fluid boundary layer theory, the flow velocity approaches zero in the liquid layer immediately adjacent to the solid interface, severely limiting intermixing between the EQZ and the FZ. Consequently, conventional alloying strategies have limited effectiveness in modulating the EQZ. Theano et al. [91] employed AA4047 filler wire with high silicon content (12 wt.%) during welding of AA2198 Al-Li alloy and reported that, although almost no silicon was detected within the EQZ, the use of the filler wire still reduced the EQZ width compared to autogenous welding (Figure 6b). Liu et al. [71] reported that reducing the laser welding heat input from approximately 60.8 kJ/m to 56.1 kJ/m and further to 46.31 kJ/m resulted in a corresponding decrease in EQZ width from 71.53 μm to 53.14 μm and finally to 31.05 μm. Similarly, Gutierrez et al. [87] reported consistent findings in their TIG welding experiments on 2195 Al-Li alloy. They observed that as the heat input decreased from 2.62 kJ/m to 0.2 kJ/m, the EQZ width exhibited a substantial reduction from 600 μm to 20 μm; concurrently, the grain size within the EQZ also decreased from approximately 40 μm to 6 μm. Zhou et al. [92] introduced beam oscillation during welding of 2060 Al-Li alloy and found that, although oscillation refined the grains within the EQZ through enhanced molten pool stirring, it unexpectedly led to an expansion of the EQZ width (Figure 6c). Experiments conducted by D. K. Aidun et al. [93] demonstrated that under hypergravity conditions (1 g → 5 g → 10 g), the EQZ progressively diminished and eventually disappeared completely in 2195-T8 Al-Li alloy welds. This phenomenon was attributed to the breakdown of the stagnant boundary layer and the convective transport of heterogeneous nucleation particles, such as Al_3_Zr, into the high-temperature region of the weld pool, where they dissolved, thereby suppressing EQZ formation. Although various strategies for EQZ control, including thermal input management, alloy composition optimization, and external field intervention, have been investigated, a processing method that is both highly efficient and practically viable for industrial applications remains unavailable.

**Figure 7:**
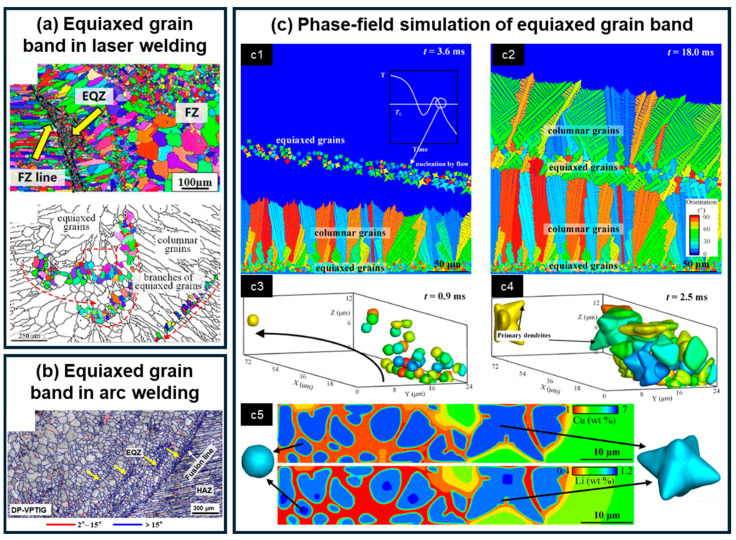
Equiaxed grain bands in Al-Li alloy weld: (**a**) Laser weld [34]; (**b**) Arc weld [37]; (**c**) Formation process of equiaxed fine grain bands reproduced by phase-field simulation, with (**c1**,**c2**) and (**c3**–**c5**) representing the 2D and 3D views, respectively. [86].

In addition to the EQZ located adjacent to the fusion line, bands of fine equiaxed grains with a morphology similar to that of the EQZ may also form within the FZ. Figure 7a,b illustrates such bands observed in both laser and arc welds of Al-Li alloys. It should be emphasized that the formation mechanism of these bands differs from that of the EQZ along the fusion boundary. Han et al. [77,86,94] systematically investigated the nucleation mechanism of these equiaxed bands using the overlap welding method originally proposed by Kou [95] for identifying weld nucleation mechanisms. Their results indicate that the formation of these bands is consistent with a grain detachment mechanism. Through thermal cycle simulations, Han et al. demonstrated that temperature fluctuations induced by thermal convection in the mushy zone of the weld pool cause partially solidified grains to detach from the solid–liquid interface. These detached grains are subsequently transported into the interior of the weld pool by fluid flow, where they serve as nucleation sites, increasing the nucleation rate and ultimately forming bands of fine equiaxed grains aligned with the flow trajectory. This mechanism was further validated through simulations of individual grain detachment and motion [86]. In addition to detached grains, high-melting-point particles, such as Al_3_Zr, with melting temperatures around 1853 K [96], may also be transported into the weld pool and promote heterogeneous nucleation in cooler regions. As shown in Figure 7a,b, the equiaxed grain bands are more pronounced in arc welding compared to laser welding, which may be attributed to the relatively lower temperature of the arc weld pool. This condition allows both detached grains and Al_3_Zr particles to survive and act as effective nucleation sites. Han et al. successfully reproduced the formation process of these equiaxed bands in Al-Li alloys through numerical simulation, as shown in Figure 7c, providing further support for the proposed mechanisms.

### 4.2. Columnar Grain Zone (CGZ)

During solidification of Al-Li alloy welds, a high thermal gradient in the molten pool promotes the preferential growth of crystals as columnar grains along the direction of maximum heat flow, typically perpendicular to the fusion line and oriented toward the weld center. Grains whose <100> close-packed directions are closely aligned with the heat flow direction exhibit a growth advantage, whereas those with larger misorientations are gradually suppressed, ultimately resulting in the formation of a coarse and highly oriented columnar grain zone. This microstructure leads to anisotropic mechanical properties, as columnar grain boundaries frequently act as preferred paths for crack propagation, thereby reducing weld strength and toughness [39,97,98,99]. In addition, the coarse columnar structure induces stress concentration, accelerates fatigue crack initiation, and deteriorates the fatigue performance of the weld. More importantly, columnar grains substantially influence the weldability of Al-Li alloys [38].

Chen et al. [100] developed a model integrating the phase-field method and computational fluid dynamics to predict the hot cracking susceptibility associated with columnar grains in Al-Li alloys. The simulation results indicated that liquid channels at the dendritic tips of columnar grains are significantly narrower than those at the roots. This inhomogeneous channel geometry is identified as a primary cause of feeding difficulties during solidification. Such geometric characteristics generate a pronounced pressure drop along grain boundaries, imposing substantial resistance to liquid metal flow and severely impairing feeding capacity [101,102]. Moreover, misorientation between adjacent columnar grains modifies the morphology and tortuosity of intergranular channels, further increasing susceptibility to solidification cracking. Zhao et al. [38] compared welding of 2195 Al-Li alloy performed without filler wire and with ER2319 filler wire, and found that cracks propagated preferentially along columnar grain boundaries. Their study further revealed that during solidification, columnar grains create long and tortuous liquid feeding channels, which hinder liquid metal backfilling and result in microvoid formation due to insufficient feeding, thereby initiating cracks. Simultaneously, wide precipitation-free zones and segregation of brittle impurity phases readily form between columnar grains, severely weakening grain boundary strength. Under solidification shrinkage stress, cracks readily initiate and propagate along these weakened boundaries. Consequently, current research on welding of Al-Li alloys frequently aims to eliminate or suppress the formation of columnar grains.

In the authors’ previous work [27], the CET behavior in Al-Li alloy welds were systematically investigated, and a CET criterion for TIG welding was proposed as $\varphi =1-exp\left[\left(C_{1}\right)\times 10^{7}\pi N_{0}\left(V^{C_{2}}/G^{3}\right)\right]$, where φ represents the fraction of equiaxed grains. Specifically, φ < 0.66% corresponds to a fully columnar structure, 0.66% < φ < 49% indicates a mixed grain structure, and φ > 49% denotes a fully equiaxed structure. $C_{1}$ and $C_{2}$ are constants, $N_{0}$ is the nucleation site density, $N_{0}$ is the temperature gradient, and V is the solidification rate. It can be observed that the weld grain morphology is jointly determined by the nucleation site density, temperature gradient, and solidification rate. Once the welding method is selected, the temperature gradient and solidification rate are largely fixed, with limited adjustability. For example, both G and V are significantly higher in laser welding than in TIG welding, making it difficult to align these parameters merely by adjusting process conditions. In contrast, the nucleation site density ($N_{0}$) is highly tunable. A higher nucleation density increases the nucleation rate, and when newly formed equiaxed grains impinge on growing columnar grains, they hinder further columnar growth. Therefore, the suppression of columnar grains and the promotion of equiaxed grain formation and grain refinement are achieved simultaneously. Currently, the most widely adopted strategy for controlling columnar grains involves promoting equiaxed crystallization by increasing the number of nucleation sites within the molten pool. The underlying principle is that newly formed grains ahead of the columnar growth front can impede further epitaxial growth of columnar grains through grain boundary interactions, thereby refining the overall grain structure. Consequently, introducing a high density of effective nucleating particles can substantially suppress columnar grain formation.

**Figure 8:**
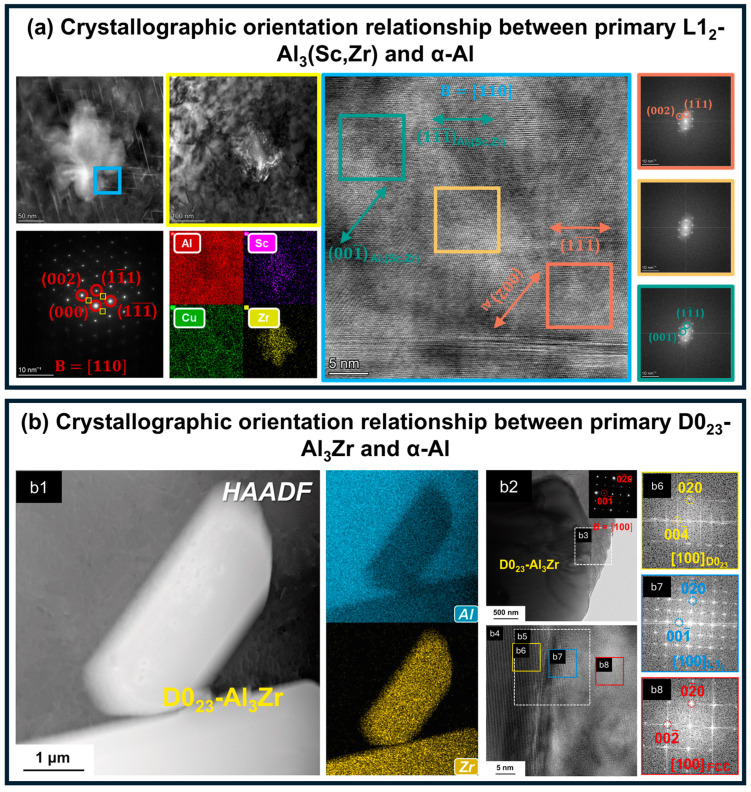
Nucleation Sites in Al-Li Alloys: (**a**) Primary L1_2_-Al_3_(Sc,Zr) phase in Al-Li alloy weld [27]; (**b**) Primary D0_23_-Al_3_Zr phase in cast Al-Li alloy, with (**b1**) showing the morphology of D023-Al_3_Zr, (**b2**–**b5**) displaying the Al3Zr/α-Al interfaces, and (**b6**–**b8**) presenting the corresponding Fast Fourier Transform (FFT) patterns [105].

According to Kou’s theory [95], nucleation mechanisms in weld metals primarily include heterogeneous nucleation, dendrite fragmentation, and grain detachment. Among these mechanisms, grain detachment provides limited refinement effectiveness due to the difficulty in supplying a sufficient number of stable nuclei, and the detached grains may remelt in the high-temperature pool, rendering this approach unreliable. Currently, the principal methods for increasing nucleation rates and inhibiting columnar growth are heterogeneous nucleation and dendrite fragmentation. Commonly employed heterogeneous nucleation agents in Al-Li alloy welds include TiC particles, primary Al_3_(Sc,Zr), and primary Al_3_Zr. Their effectiveness as nucleation sites arises from their crystallographic similarity to α-Al, which requires only minimal undercooling to activate and provides potent substrates for α-Al solidification. These particles exhibit low lattice misfit with α-Al, with Al_3_Zr displaying a misfit of only 0.5 percent [103] and Al_3_(Sc,Zr) less than 1.5 percent [104]. In addition, they maintain well-defined crystallographic orientation relationships with α-Al. As shown in Figure 8a, the L1_2_-Al_3_(Sc,Zr) phase serves as an active nucleation site in Al-Li welds, with the TEM-derived orientation relationship described as follows [27]: ${\left[211\right]}_{\alpha -Al}∥{\left[211\right]}_{{Al}_{3}\left(Sc,Zr\right)}$, ${\left(1\bar{1}\bar{1}\right)}_{\alpha -Al}∥{\left(1\bar{1}\bar{1}\right)}_{{Al}_{3}\left(Sc,Zr\right)}$, ${\left(02\bar{2}\right)}_{\alpha -Al}∥{\left(01\bar{1}\right)}_{{Al}_{3}\left(Sc,Zr\right)}$, ${\left[110\right]}_{\alpha -Al}∥{\left[110\right]}_{{Al}_{3}\left(Sc,Zr\right)}$, and ${\left(002\right)}_{\alpha -Al}∥{\left(001\right)}_{{Al}_{3}\left(Sc,Zr\right)}$. Similarly, Figure 8b shows the orientation relationship between D0_23_-Al_3_Zr and α-Al [105]: ${\left[110\right]}_{\alpha -Al}∥{\left[110\right]}_{{Al}_{3}Zr}$, ${\left(00\bar{2}\right)}_{\alpha -Al}∥{\left(00\bar{4}\right)}_{{Al}_{3}Zr}$.

**Figure 9:**
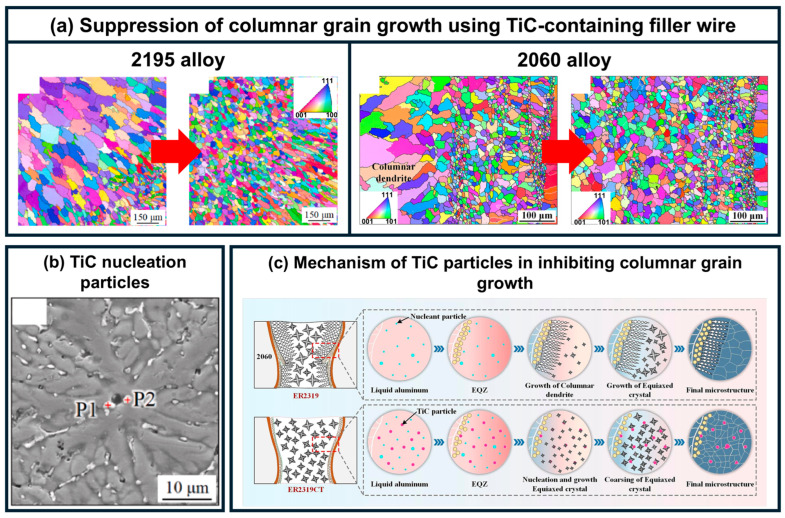
Effect of TiC-containing filler wire on columnar grains in Al-Li alloy weld [106,107]: (**a**) Macrostructural suppression of columnar grains; (**b**) Morphology of TiC nucleation particles; (**c**) Schematic of grain refinement mechanism.

As illustrated in Figure 9, studies conducted by Zhan et al. [106] and Li et al. [107] demonstrated that the introduction of TiC nanoparticles during laser welding of Al-Li alloys significantly inhibits columnar grain growth and promotes the formation of equiaxed grains. The addition of TiC reduced the average grain size in the weld by approximately 47 percent and mitigated the basal texture tendency. The refined microstructure resulted in substantial improvements in mechanical properties: under optimal processing conditions, the ultimate tensile strength reached 318 MPa, and the elongation nearly doubled. Fracture analysis indicated that TiC particles enhance toughness by promoting crack deflection and hindering dislocation motion.

**Figure 10:**
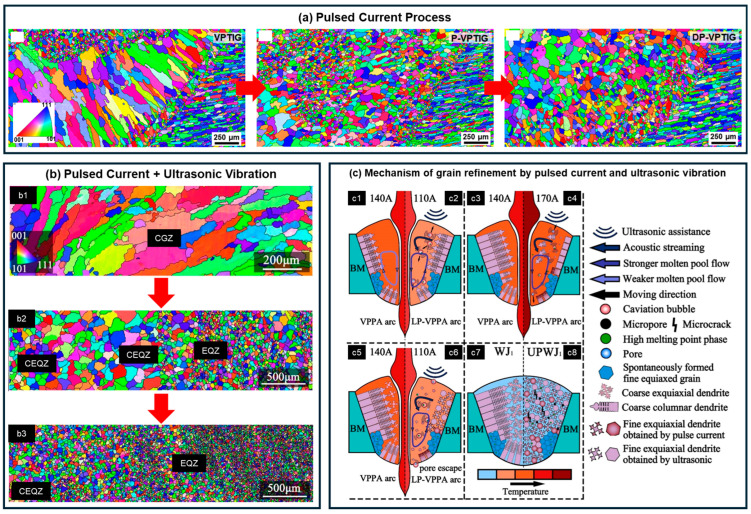
Effect of welding process on columnar grains in Al-Li alloy weld: (**a**) Low-frequency pulsed current and low-frequency + ultrasonic-frequency pulsed current [37]; (**b**) Pulsed current + ultrasonic vibration process, with (**b1**) conventional welding, (**b2**) ultrasonic vibration-assisted welding, and (**b3**) pulsed current + ultrasonic vibration-assisted welding (CEQZ: central equiaxed grain region; EQZ: equiaxed grain zone; CGZ: columnar grain zone) [71]; (**c**) Mechanism of grain refinement by pulsed current and ultrasonic vibration [71], where (**c1**,**c3**,**c5**) represent conventional welding, (**c7**) shows the final microstructure in conventional welding, (**c2**,**c4**) correspond to the background current stage and peak current stage in pulsed current + ultrasonic vibration-assisted welding respectively, (**c6**) shows the next background current stage, and (**c8**) presents the corresponding final microstructure.

Han et al. [36] employed a laser beam oscillation process in combination with a Zr-containing filler wire, which facilitated the formation of L1_2_-Al_3_Zr heterogeneous nucleation particles during rapid solidification. This approach increased nucleation rates, suppressed the growth of coarse columnar grains, and refined the grain size from approximately 100 μm to around 7 μm, while increasing the tensile strength from 228 MPa to 379 MPa. Sun et al. [37] implemented a hybrid pulsed current process combining low and ultrasonic frequencies to apply pulsating impacts on the molten pool. This treatment fragmented growing dendrites and introduced new nuclei, thereby eliminating coarse columnar structures and refining grains in the FZ (Figure 10a). Cui et al. [71] initially applied a low-frequency pulsed current to refine columnar grains from approximately 200 μm to equiaxed grains of roughly 50 μm, and subsequently combined this treatment with ultrasonic vibration to further reduce the grain size to about 20 μm (Figure 10b). The refinement was attributed to ultrasonic cavitation-induced dendrite fragmentation and nucleation, accompanied by acoustic streaming that promoted uniform distribution of cavitation bubbles throughout the FZ, resulting in homogeneous microstructural refinement. In a separate study, Cui et al. [83] also employed Zr- and Ti-rich filler wires to inhibit the formation of coarse columnar grains in welds. Ti, with its high growth restriction factor, effectively limited grain growth in the FZ, thereby contributing to grain refinement in Al-Li alloy welds. Table 4 categorizes grain refinement mechanisms in weld seams, lists corresponding microstructural characteristics, and provides a comparative summary of current grain refinement strategies and their effectiveness in Al-Li alloy welds.

**Figure 11:**
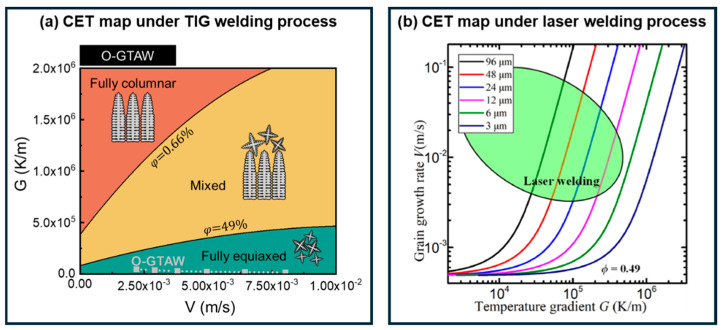
Columnar-to-equiaxed transition (CET) maps in Al-Li alloy welds: (**a**) CET map for TIG-welded cast Al-Li alloy Al-2Cu-2Li-0.5Mg-0.2Sc-0.15Zr [27]; (**b**) CET map for laser-welded 2195 Al-Li alloy [86].

To gain a deeper understanding of the columnar-to-equiaxed transition (CET) phenomenon in Al-Li alloy welds, Li et al. [27] and Han et al. [86] investigated the CET process and developed CET maps based on Hunt’s model, as shown in Figure 11. These maps enable quantitative prediction of the relative proportions of columnar and equiaxed grains under specific welding process parameters. Han et al. [86] plotted CET diagrams for varying initial nucleation densities using material parameters referenced from Rebow et al. [108] (Figure 11b). The green box indicates the range of thermal gradients and crystal growth rates typical of laser welding processes. Their results demonstrate that at high initial nucleation densities, corresponding to finer grain sizes (for example, curves representing 6 μm and 3 μm in Figure 11b), fully equiaxed grain structures can be achieved throughout the entire weld under all laser welding conditions. Li et al. employed the KGT model [109] to calculate and fit material parameters for a specific Al-Li alloy composition and subsequently constructed a CET map for this alloy. The presence of a high density of primary L1_2_-Al_3_(Sc,Zr) particles ensured that welds produced by TIG welding, indicated by the gray dashed line in Figure 11a, consistently fell within the fully equiaxed zone. Studies on CET in Al-Li alloy welds confirm that a high density of nucleation sites in the molten pool can effectively suppress the formation of columnar grains induced by steep thermal gradients during welding.

## 5. Prospects and Future Work

Despite significant advances in understanding the weldability and microstructural evolution of Al-Li alloys, several challenges remain before their full potential can be realized in aerospace and other demanding applications.

The equiaxed zone (EQZ) microstructure remains a major obstacle to producing high-quality joints in Al-Li alloy welds. Although its formation mechanisms have been extensively studied, effective approaches to suppress its formation or alleviate its detrimental effects are still lacking. A comprehensive understanding of cracking within the EQZ requires the development of advanced numerical models, particularly coupled phase-field and stress-field solidification models, to clarify crack initiation mechanisms. Since the EQZ generally contains few strengthening precipitates, its presence significantly compromises mechanical performance. Nevertheless, post-weld heat treatment can facilitate solute back-diffusion and promote precipitate formation, thereby improving the overall mechanical properties. However, investigations into precipitate evolution and the associated mechanical response of the EQZ under different heat treatment conditions remain limited. Future work should therefore emphasize the characterization of precipitates in the as-welded EQZ under various welding parameters, as well as the monitoring of their transformation during subsequent post-weld heat treatments.

Although substantial progress has been achieved in refining weld grain structures and promoting the columnar-to-equiaxed transition (CET), the economic feasibility of these strategies remains a critical consideration for industrial application. Alloying additions such as Sc and Zr, which are highly effective grain refiners, are prohibitively expensive. Meanwhile, process-based approaches, including beam oscillation and ultrasonic assistance, face challenges associated with high equipment costs and potential degradation of weld formation quality. Furthermore, existing refinement mechanisms in Al-Li welds are largely based on heterogeneous nucleation, while comparatively little attention has been directed toward enhancing melt pool undercooling or employing growth restriction strategies. Future research could investigate the use of cost-effective TiB_2_ refiners, which are already commercially established for conventional aluminum alloys. In addition to the heterogeneous nucleation provided by TiB_2_ particles, the high growth restriction factor of solute Ti itself may further enhance grain refinement in welded joints.

As precipitation-strengthened alloys, the strength and joint efficiency of Al-Li alloys are largely dependent on their nanoscale precipitates [110], yet they have been examined only to a limited extent with respect to direct artificial aging after welding, while comprehensive post-weld heat treatments, including solution treatment followed by aging, remain relatively underexplored. Future studies should focus on developing tailored post-weld heat treatment protocols specifically designed for Al-Li alloy welds. Particular attention should be given to optimizing treatments for the heterogeneous grain structures characteristic of welded joints, which typically include the wrought base metal, the EQZ, and the columnar grain zone.

Addressing these issues through coordinated experimental and computational efforts will provide the scientific foundation for reliable welding of next-generation lightweight structures.

## Figures and Tables

**Table 1:** Comparison of composition and welding behavior across Al-Li alloy generations.

Generation	Representative Alloy Systems	Composition	Weldability	Microstructural Characteristics	Welding Defects
1st	Al-Mg-Li system (e.g., 1420)	High Li, Mg; minor Sc, Zr	Moderate; Mg reduces cracking, Li increases porosity	Equiaxed grains preferred	High porosity; reduced cracking
2nd	Al-Cu-Li system (e.g., 2090, 1430, 1440, 8090, 1460)	1–3 wt.% Cu; >2 wt.% Li; trace Sc	Poor; prone to solidification cracking and H porosity	Equiaxed grains preferred	Severe porosity; cracking along Cu-rich zones
3rd	Advanced Al-Cu-Li-(Mg, Ag, Zn) systems (e.g., 2094, 2095, 2195, 2197, Airware™ 2196/2198, Weldalite™ 049/210)	Higher Cu/Li; trace Mg, Ag, Zn	Improved; better strength-ductility balance	Columnar grains preferred	Low porosity; crack-resistant

**Table 2:** Comparison of simulation methods used in Al-Li alloy welding.

Material	Simulation Method	Model Assumptions	Application	Main Findings
Al-Li alloy	Computational Fluid Dynamics	Continuum medium; Gaussian heat source; laminar Newtonian flow	Simulation of weld pool flow, temperature distribution, and keyhole oscillation behavior	Melt flow–induced grain detachment promotes equiaxed grain formation [77]
	Phase-Field Method	Multi-component dilute solution model; diffuse interface; crystallographic anisotropy	Simulation of nucleation, growth, and solute redistribution during solidification	Predicted columnar-to-equiaxed transition (CET) and solute segregation behavior [80]
	Finite Element Method	T-joint configuration; sequential thermomechanical coupling; double-ellipsoidal Gaussian heat source	Simulation of residual stress and deformation in specific welded structures	High compressive stress concentrated near T-joint weld toes [78]

**Table 3:** Comparative assessment of welding techniques on joint efficiency, porosity control, and process characteristics.

Welding Process	Technique	Joint Efficiency	Porosity Control	Key Advantage	Possible Limitation
Laser welding	Heat input control [72]	70.62–82.58%	with a porosity of 1.83–2.78%	Easily adjustable	Limited efficacy
	Hybrid heat source [73]	-	with a porosity of 0.7–4.51%	Stable molten pool; negligible porosity	Requires specialized equipment
	Beam oscillation [36]	49.04–65.91%	Observable reduction	Effective grain refinement	Requires specialized equipment
Arc welding	Back gas-shielded [85]	62.45%	Significantly reduced porosity	Easily adjustable	Limited efficacy for partial penetration
	Pulsed current [27]	90.09–91.58%	Observable reduction	Easily adjustable	Limited efficacy
	Ultrasonic vibration assistant [71]	48.24–57.36%	Observable reduction	Effective grain refinement	Requires specialized equipment

**Table 4:** Comparison of grain refinement mechanisms, corresponding microstructural characteristics, and implementation approaches in Al-Li alloy welds.

Refinement Mechanism	Weld Microstructure Characteristics	Implementation Approach	Resulting Grain Size
Grain Detachment	Formation of bands of fine equiaxed grains aligned with the weld pool flow trajectory	High-speed fluid flow within the weld pool	Locally refined
Heterogeneous Nucleation	Formation of fine equiaxed grains, promoting the columnar-to-equiaxed transition	Filler wire containing TiC nanoparticles; Use of filler wire or base metal containing Sc or Zr	Significantly refined
Dendrite Fragmentation	Formation of fine equiaxed grains, promoting the columnar-to-equiaxed transition	Beam oscillation; Ultrasonic vibration assistance; Pulsed current process	Significantly refined

## Data Availability

No new data were created or analyzed in this study. Data sharing is not applicable to this article.

## Abbreviations

EQZ	Equiaxed grain zone
DLBW	Dual laser beam welding
TIG	Tungsten inert gas
PMZ	Partially melted zone
FZ	Fusion zone
CGZ	Columnar grain zone
VPPA	Variable polarity plasma arc
HAZ	Heat-affected zone
BM	Base metal
